# Multidimensional Factors Related to Engagement in Child-to-Parent Violence among Adolescents with Attention-Deficit/Hyperactivity Disorder

**DOI:** 10.7150/ijms.116091

**Published:** 2025-09-03

**Authors:** Wen-Jiun Chou, Ray C. Hsiao, Peng-Wei Wang, Cheng-Fang Yen

**Affiliations:** 1 Department of Child and Adolescent Psychiatry, Chang Gung Memorial Hospital, Kaohsiung Medical Center, Kaohsiung, Taiwan.; 2 College of Medicine, Chang Gung University, Taoyuan, Taiwan.; 3 Department of Psychiatry, Seattle Children's, Seattle, WA, USA.; 4 Department of Psychiatry and Behavioral Sciences, School of Medicine, University of Washington, Seattle, WA, USA.; 5 Department of Psychiatry, Kaohsiung Medical University Hospital, Kaohsiung, Taiwan.; 6 Department of Psychiatry, School of Medicine College of Medicine, Kaohsiung Medical University, Kaohsiung, Taiwan.; 7 College of Professional Studies, National Pingtung University of Science and Technology, Pingtung, Taiwan.

**Keywords:** attention-deficit/hyperactivity disorder, behavior problems, child-to-parent violence, domestic violence, parenting styles, social information processing.

## Abstract

**Background:** Child-to-parent violence (CPV) has received increasing attention because of the growing response from parents. This study examined the rates of various CPV types and the reasons underlying why adolescents with attention-deficit/hyperactivity disorder (ADHD) engage in CPV and examined the associations of multidimensional factors with CPV in adolescents with ADHD.

**Method:** In total, 247 adolescents with ADHD and their parents participated in the study. The types (including psychological aggression, physical aggression, financial demand, and control or domination) and instrumental and reactive reasons of CPV, individual factors (demographic characteristics, ADHD symptoms, internalizing and externalizing behavior problems, self-esteem, and social information processing [SIP] during conflict), parent-child interaction factors (parenting styles and parent-to-child violence), and family factors (violence among adult family members) were collected.

**Results:** In total, 78.9% of adolescents with ADHD engaged in CPV in the year preceding their evaluation. Both instrumental and reactive reasons were reported for CPV. Aggression response of SIP (*p* = .003) and parent-to-child violence (*p* < .001) were positively associated with psychological aggression. Externalizing behavior problems were positively associated with physical aggression (*p* < .001) and financial demand (*p* = .001). Finally, externalizing behavior problems (*p* = .001), aggression response of SIP (*p* = .001), and violence among adult family members (*p* = .006) were positively associated with control or domination.

**Conclusion:** Many adolescents with ADHD had engaged in CPV. Multiple individual, parent-child interaction, and family factors were significantly correlated with CPV.

## Introduction

Child-to-parent violence (CPV) has received increasing attention because of the growing response from parents 1. In a review of relevant studies, Simmons et al. reported that approximately 5% to 21% of children exhibited a certain form of physical aggression toward their parents among the general population 2. In a systematic review and meta-analysis, Burgos-Benavides et al. indicated that 23% to 25% of children and adolescents in Latin America returned to psychological violence and 5% to 6% returned to physical violence against their parents 3. In a study conducted in Australia, Simmons et al. reported that 7% of the respondents (aged 14 to 25 years) had physically attacked their parents 4. These findings suggest that adolescent attacks against parents are not uncommon.

CPV is a topic that necessitates further exploration for several reasons. First, adolescent-to-parent violence can be a precursor to various forms of violent crime 5. A compilation of crime cases in Spain revealed that adolescents who attack their parents are often the most serious offenders among minors 1. Second, adolescent-to-parent violence directly causes loss of parental discipline and feelings of humiliation, which may compromise the safety of family members 6-8.

Involvement in violence is a major concern for adolescents with attention-deficit/hyperactivity disorder (ADHD). In a systematic review, Buitelaar et al. reported that adolescents with ADHD are at a higher risk of domestic and intimate partner violence during adulthood compared with those without ADHD 9. Despite these findings, few studies have examined CPV in adolescents with ADHD. In a study conducted in the United States, Edwards et al. discovered that boys with comorbid ADHD and oppositional defiant disorder (ODD) were more likely than their healthy counterparts to engage in aggressive conflict with their parents 10. In a study on Iranian parents of adolescents with ADHD, Ghanizadeh and Jafari reported that more than half of these parents had experienced at least one physical, verbal, psychological, or materialistic attack by their children, with comorbid ODD, tic disorder, separation anxiety, and maternal depression serving as factors related to CPV 11. In a study on Japanese adolescents receiving pharmacological treatment for ADHD, Sasaki et al. discovered that receiving three or more types of medication for ADHD was significantly correlated with CPV 12.

According to ecological systems theory 13, CPV in adolescents with ADHD is the unique result of individual-microsystem interactions that may differ from those of adolescents without ADHD. Compared with adolescents without ADHD, those with ADHD tend to have greater cognitive dysfunction 14, more types of information processing 15, and higher engagement in risk-taking behaviors 16. Adolescents with ADHD may also experience unique forms of parent-child conflict 10, 17 and unique parenting styles 18. In addition, they are more likely than other family members to experience domestic violence 19. These factors may contribute to the development of parent-child conflict and increase the risk of CPV in adolescents with ADHD. Therefore, further research is required to examine the factors related to CPV in adolescents with ADHD.

Studies have identified several individual and parent-child interaction factors that correlate with CPV among the general population of adolescents. Verbal and psychological CPV are more common in girls 20, whereas physical CPV is more common in boys 21. Adolescents who engage in CPV are more likely than other adolescents to exhibit ODD, ADHD, depression, impulsivity, and deviant behavior 20-22. Hostile attribution and anger, two types of social information processing, can predict CPV 1 year later in both boys and girls 23. Some studies have suggested that adolescents with low self-esteem are at an increased risk of engaging in CPV 21, whereas others have indicated that those with high self-esteem are at an increased risk of attacking their parents 22. In terms of parent-child interaction factors, poor parent-child communication 24, 25, insufficient parental discipline 22, 24, parental discipline through punishing and yelling 26, and lack of parental warmth toward children 27 are significantly correlated with CPV. However, the associations between these individual or parent-child interaction factors and CPV in adolescents with ADHD require further analysis.

This study had two goals. First, we examined the rates of various CPV types and the reasons underlying why adolescents with ADHD engage in CPV. Second, we examined the associations of individual factors (demographic characteristics, ADHD symptoms, internalizing and externalizing behavior problems, self-esteem, and social information processing during conflict), parent-child interaction factors (parenting styles and parent-to-child violence), and family factors (violence among adult family members) with CPV in adolescents with ADHD.

## Methods

### Participants and procedure

In this cross-sectional study, we distributed surveys to adolescents with ADHD and their primary caregivers. Adolescents with ADHD from six child psychiatry outpatient clinics at two hospitals in Taiwan were included for analysis. Adolescents with ADHD meeting the following criteria were included in the study: (1) being 11-18 years of age and (2) having received a diagnosis of ADHD by a certified child psychiatrist in accordance with the *Diagnostic and Statistical Manual of Mental Disorders, Fifth Edition* 28. Adolescents who had comorbid intellectual disability, severe autism spectrum disorder, bipolar disorder, schizophrenia, or any other cognitive deficits that may impede their understanding of the study purposes and completion of the research questionnaire were excluded, along with their parents.

Three child psychiatrists reviewed the medical records of adolescents with ADHD who visited the selected outpatient clinics between August 2023 and July 2024. A total of 259 adolescents with ADHD and their parents were consecutively approached. The child psychiatrists interviewed the adolescents and their parents and excluded 12 adolescents with ADHD because they had either a comorbid autism spectrum disorder (*n* = 6) or an intellectual disability (*n* = 6). Subsequently, the child psychiatrists explained the study purposes and procedures to the remaining adolescents and their parents and invited them to participate in the study. All participants were assured that their responses would remain confidential and that their participation or nonparticipation would not influence their right to receive medical services. In total, 247 adolescents with ADHD and their parents agreed to participate in the study.

### Ethical considerations

This study was approved by the Institutional Review Boards of Kaohsiung Medical University Hospital (KMUHIRB-SV(II)-20210113) and Chang Gung Memorial Hospital, Kaohsiung Medical Center (202102157A3C601). Informed consent was obtained from all adolescents and their parents. This study employed a survey design and did not involve experiments on humans or human tissue samples. This study was conducted in accordance with the Declaration of Helsinki and the Recommendations for the Conduct, Reporting, Editing, and Publication of Scholarly Work in Medical Journals by the International Committee of Medical Journal Editors.

### Measures

#### Child-to-parent violence questionnaire

We used the Child-to-Parent Violence Questionnaire (CPV-Q) to evaluate CPV among adolescents in the year preceding evaluation 29, 30. The CPV-Q is divided into two parts. The first part consists of 14 items that evaluate four domains of CPV, namely psychological aggression (four items, e.g., “I have told my parents 'I hate you!' and 'I wish you were dead'''), physical aggression (three items, e.g., “I have thrown things at my parents”), financial demand (three items, e.g., “I have demanded that my parents buy me things I know they cannot afford”), and control or domination (four items, e.g., “I have told my parents that at home they have to do what I want”). Each item is rated on a 5-point scale with endpoints of 0 (*never*), 1 (*once*), 2 (*two to three times*), 3 (*four to five times*), and 4 (*six times or more*). The second part consists of eight items evaluating two main categories of reasons for CPV, namely instrumental reasons (e.g., “To be able to come home later when going out at night”) and reactive reasons (e.g., “In response to a parents' physical aggression”). In this study, we added another four reasons for CPV according to our clinical experience, namely in response to parents' restricting their children's use of 3C products, in response to parents' different treatment styles with their children, in response to parents' restricting their children's social communication or intimate relationships, and in response to parents' control of how their children dress. Each reason was rated on a 4-point scale with endpoints ranging from 0 (*never*) to 3 (*always*).

Adolescents and their parents were invited to complete the CPV-Q. Both versions of the questionnaire had the same content but differed only in their phrasing style. For example, the third item was phrased as “I have made negative, offensive, and/or degrading comments to my parents” in the child version but as “My child has made negative, offensive, and/or degrading comments to me” in the parent version. The CPV-Q has acceptable reliability and validity 31. In a study comparing 11 instruments for evaluating CPV based on the COSMIN guidelines, Ibabe concluded that the CPV-Q was of high quality 1. In the present study, the internal consistency (McDonald's ω) of the four domains of both CPV-Q versions ranged from 0.65 to 0.78, and the Pearson's correlation coefficient for the 2-week test-retest reliability of both versions ranged from 0.62 to 0.73 (*p* < .001). Adolescents whose answers were not 0 to any item in the child version of the CPV-Q were regarded as having CPV. Similarly, adolescents whose parents' answers were not 0 to any item in the parent version of the CPV-Q were regarded as having CPV.

#### Social information processing in child-to-parent conflicts questionnaire

We used the Social Information Processing in Child-to-Parent Conflicts Questionnaire (SIPCPC-Q) to evaluate adolescents' self-reported social information processing during child-to-parent conflict 23. Adolescents were asked to imagine three scenarios involving conflict with their parents. For each scenario, they were asked to rate seven items on a 5-point scale with endpoints ranging from 0 (*not at all*) to 4 (*to a great extent*). These items evaluated the following five social information processing components: hostile attribution, including the attribution of negative intentions or positive emotions to parents (two items per scenario); anger (one item per scenario); aggressive response, including both physical and psychological aggression (two items per scenario); anticipation of positive personal outcomes due to aggressive action, such as the conferral of privileges or the avoidance of unpleasant tasks (one item per scenario); and empathy or anticipation of negative consequences for parents due to aggressive actions (one item per scenario). The SIPCPC-Q has acceptable reliability and validity 23. In this study, the internal consistency (McDonald's ω) of the five domains of the SIPCPC-Q ranged from 0.70 to 0.82, and the Pearson's correlation coefficient for the questionnaire's 2-week test-retest reliability ranged from 0.65 to 0.78 (*p* < .001).

#### Rosenberg self-esteem scale

We used the 10-item Rosenberg Self-Esteem Scale to evaluate adolescents' self-reported self-esteem 32. Each item was rated on a 4-point scale with endpoints ranging from 1 (*strongly disagree*) to 4 (*strongly agree*), yielding a single overall self-esteem score, with high scores indicating high levels of self-esteem. This scale has been used to evaluate self-esteem levels in Taiwanese adolescents 33. In this study, the Cronbach's ⍺ of the scale was 0.860.

#### Child behavior checklist for ages 6-18

We used the 112-item parent-reported Chinese version of the Child Behavior Checklist for Ages 6-18 to evaluate adolescents' behavioral problems 34-36. We also used the recommended T-score transformations of raw behavior scores, which were adjusted for age and sex differences in behavior found in normative samples. In addition, we used the following domains for analysis ADHD symptoms, internalizing problems (evaluated using scales for anxiety/depression, withdrawal/depression, and somatic syndrome disorder), and externalizing problems (evaluated using scales for ODD and conduct symptoms). This checklist has an internal consistency (Cronbach's α) of 0.55-0.90 and a 1-month test-retest reliability (Pearson's *r*) of 0.51-0.74, along with high construct validity (eight-factor structure) 37, 38.

#### Parental bonding instrument

We used the 25-item Chinese version of the Parental Bonding Instrument, parent version, to evaluate parents' perceptions of three parenting styles: caring or affectionate parenting, authoritative parenting, and overprotective parenting 39. In this instrument, each item is rated on a 4-point Likert scale. A high score on the care/affection subscale indicates perceptions of parental warmth and affection, whereas a low score indicates perceptions of rejection or indifference. The overprotection subscale measures overprotective parenting behaviors and denial of adolescents' psychological autonomy. The authoritarianism subscale evaluates the degree of authoritative control that parents exert over their adolescents' behavior 40. The reliability and validity of the Chinese version of the Parental Bonding Instrument were established in a previous study 41. In the present study, the Cronbach's α values for caring or affectionate parenting, overprotective parenting, and authoritative parenting were 0.78, 0.70, and 0.68, respectively, indicating acceptable internal consistency.

#### Parent-to-child violence questionnaire and violence among adult family members questionnaire

We adopted seven items from the CPV-Q to develop the child-reported Parent-to-Child Violence Questionnaire (PCV-Q) and parent-reported Violence Among Adult Family Members Questionnaire (VAFM-Q). The PCV-Q was used to evaluate parental verbal (four items) and physical (three items) violence against adolescents in the year preceding evaluation. The VAFM-Q was used to evaluate verbal and physical violence among adult family members within the same timeframe. The items in both questionnaires were rated on the same 5-point scale as that used in the CPV-Q. The internal consistency (McDonald's ω) of the PCV-Q and VAFM-Q was calculated as 0.76 and 0.72, respectively. Answers other than 0 to any item of the PCV-Q or VAFM-Q were indicative of parent-to-child violence and violence among adult family members, respectively.

#### Demographic characteristics

Data on the gender and age of the adolescents and the gender, age, and educational level of their parents were collected.

### Data analysis

All statistical analyses were conducted using IBM SPSS Statistics version 24.0 (IBM, Armonk, NY, USA). Descriptive statistics (presented as means and frequencies) were used to summarize the characteristics of the study sample. A bivariable logistic regression analysis was conducted to examine the associations of individual factors (demographic characteristics, social information processing, self-esteem, ADHD symptoms, and internalizing and externalizing behavior problems), parent-child interaction factors (parenting styles and parent-to-child violence), and family factors (violence among adult family members) with four domains of CPV. Factors that exhibited a significant correlation with CPV in the bivariable logistic regression analysis were included in a multivariable logistic regression analysis to further explore their associations with CPV. The results are presented as odds ratios and 95% confidence intervals. Because of the multiple comparisons made between various factors and the four domains of CPV, a *p* value less than .0125 was considered statistically significant.

## Results

Table 1 presents the participants' demographics, behavioral problems, self-esteem, and social information processing during conflict; parenting styles and violence against children; and violence among adult family members. Adolescent- and parent-reported CPV and the reasons reported for CPV are displayed in Table 2. In total, 78.9% of adolescents with ADHD had engaged in CPV in the year preceding their evaluation, according to their self-reported (56.3%) and parent-reported (66.8%) survey results. The most common type of CPV was control or domination (63.2%), followed by psychological aggression (55.5%), financial demand (46.6%), and physical aggression (17.4%). The most common reason for CPV was in response to parental restriction of children's 3C product use (69.7%), followed by CPV due to child anger (69.2%), CPV to avoid chores (61.0%), CPV to persuade parents to buy an item (53.3%), and CPV to avoid going to school or studying (50.3%).

Table 3 presents the results of the bivariable logistic regression analysis of the factors correlated with CPV. ADHD symptoms (*p* < .001), internalizing behavior problems (*p* < .001), externalizing behavior problems (*p* < .001), anger (*p* = .003), aggressive response (*p* < .001), parent-to-child violence (*p* < .001), and violence among adult family members (*p* = .001) were positively and significantly associated with psychological aggression. In addition, ADHD symptoms (*p* = .002), externalizing behavior problems (*p* < .001), aggressive response (*p* = .003), and parent-to-child violence (*p* = .001) were positively and significantly associated with physical aggression. Moreover, ADHD symptoms (*p* < .001), internalizing behavior problems (*p* < .001), externalizing behavior problems (*p* < .001), and parent-to-child violence (*p* = .003) were positively and significantly associated with financial demand, whereas self-esteem (*p* = .003) and caring or affectionate parenting (*p* = .001) were negatively and significantly associated with financial demand. ADHD symptoms (*p* < .001), internalizing behavior problems (*p* < .001), externalizing behavior problems (*p* < .001), anger (*p* = .003), aggressive response (*p* < .001), parent-to-child violence (*p* = .001), and overprotective parenting (*p* = .003) were positively and significantly associated with control or domination.

The factors significantly correlated with CPV were entered into multivariable logistic regression models (Table 4). The results indicated that aggression response (*p* = .003) and parent-to-child violence (*p* < .001) were positively and significantly associated with psychological aggression. In addition, externalizing behavior problems (*p* < .001) were positively and significantly associated with physical aggression. Moreover, externalizing behavior problems were positively and significantly associated with financial demand (*p* = .001). Finally, externalizing behavior problems (*p* = .001), aggression response (*p* = .001), and violence among adult family members (*p* = .006) were positively and significantly associated with control or domination.

## Discussion

In this study, the results indicated that 78.9% of adolescents with ADHD engaged in CPV in the year preceding their evaluation. Both instrumental and reactive reasons were reported for CPV among these adolescents. Multidimensional factors, including individual (externalizing behavior problems and social information processing involving aggressive response), parent-child interaction (parent-to-child violence), and family (violence among adult family members) factors, were found to be significantly correlated with CPV, supporting the use of ecological system theory to understand CPV.

More than three-fourths of adolescents with ADHD who participated in this study had engaged in CPV. Nearly two-thirds of them exhibited control or domination behaviors toward their parents. Even instances of the least prevalent form of CPV, namely physical aggression, were reported by 17.4% of adolescents with ADHD or their parents in the year preceding their evaluation. Overall, the rate of CPV among adolescents with ADHD observed in this study is higher than the rates reported in previous studies on the general adolescent population 2-4. Our findings support the prevalence of CPV among adolescents with ADHD. Given the negative influence of CPV on adolescents and their parents 6-8, health-care professionals should routinely screen for CPV among adolescents with ADHD and assist adolescents and parents in avoiding such behaviors.

In this study, adolescents with ADHD and their parents reported multiple reasons for engagement in CPV. The two most common reactive reasons for CPV were in response to parental restriction on adolescents' 3C product use and adolescents' anger. Problematic smartphone, internet, and screen use is prevalent among adolescents with ADHD 42, 43. Parental control over 3C product use often provokes strong resentment in adolescents with ADHD who seek instant gratification 44-47. Communication skill deficits can impede calm discussion of rules for 3C use between adolescents with ADHD and parents 48. These characteristics may lead adolescents with ADHD to engage in CPV as a means of expressing dissatisfaction and fighting for their right to use 3C products when faced with parental control. Impairing irritability is highly prevalent among individuals with ADHD 49, suggesting that outbursts of anger during parent-child interactions may lead to CPV.

In this study, the three most common reasons for adolescent engagement in CPV were to avoid chores, to persuade parents to buy a desired item, and to avoid attending school or studying. People with ADHD are highly prone to boredom, causing difficulties in activities that require sustained attention and academic performance 50, 51, thereby increasing their resistance to doing chores, attending school, and studying. CPV may result from a failure in parent-child communication regarding responsibility for chores, attending school, or studying. If CPV successfully prevents adolescents from doing chores, attending school, or studying, they may repeatedly engage in CPV. Similarly, they may engage in CPV to force their parents to buy certain items.

We observed that externalizing behavior problems, including ODD and conduct problems, were significantly correlated with physical aggression, financial demand, and controlling behavior toward parents. ODD and conduct problems may cause adolescents to neglect family, school, and social rules, thereby creating opportunities for parents to manage their adolescents' behaviors. ODD and conduct problems may also complicate parent-child communication, increasing the likelihood of conflict and CPV. Alternatively, CPV may be a manifestation of ODD or conduct problems in adolescents.

The SIPCPC-Q measures social information processing involving aggressive response through items that evaluate whether adolescents would shout at, insult, hit, or otherwise hurt their parents in parent-child conflict scenarios. Forms of social information processing that involve aggressive response may cause adolescents with ADHD to engage in CPV. Alternatively, both aggression response of SIP and CPV may also share origins in the adolescent's cognitive and emotional control difficulties.

In this study, parent-to-child violence and violence among adult family members were significantly correlated with CPV among adolescents with ADHD. Adolescents may engage in CPV to protect themselves or others from parent-to-child violence or violence among adult family members 1. Exposure to family violence can result in negative perceptions and expectations of social relationships 31, further increasing the risk of CPV during parent-child interactions 2. Adolescents who witness family violence may learn how to engage in violence as a problem-solving method 52. However, the cross-sectional study design limited the inference of the temporal relationships between parent-to-child violence, violence among adult family members and CPV. Such various types of domestic violence may have their origins in overall family dysfunction.

In the present study, we noticed that ADHD symptoms and internalizing behavior problems were significantly correlated with CPV in bivariable regression analysis. However, these associations became nonsignificant when externalizing behavior problems were also entered into the multivariable regression models. A review study indicated that comorbid conduct problems and antisocial personality disorder mediate the predictive relationship between ADHD and domestic violence 9. In the present study, self-esteem and caring or affectionate parenting exhibited a significant negative correlation with CPV in bivariable regression analysis. Although these associations became nonsignificant in multivariable regression analysis, other studies have reported that self-esteem and caring or affectionate parenting can protect adolescents from perpetrating or experiencing violence 53, 54. It is necessary to boost adolescents' self-esteem and help parents develop affectionate parenting.

In this study, most of the adolescents with ADHD were male (83.4%). In clinically referred populations, males are estimated to suffer from ADHD 2:1 to 9:1 compared to females 55, 56. However, sampling of nonreferred populations suggests the incidence may not differ, but rather reflects lower clinical referrals in females 57. No gender differences in CPV were found in this study; however, further study is needed to examine gender differences in CPV in a nonreferred population of adolescents with ADHD.

This study has several limitations. First, adolescents with ADHD were recruited from outpatient clinics, where they were actively receiving pharmacological or psychological therapy. Children with ADHD who seek treatment in medical units may have more severe ADHD symptoms than those who do not, thereby having a high rate of CPV. Alternatively, parents who bring their children to medical units for treatment of ADHD may receive advice from healthcare professionals on how to improve parent-child interactions, thereby reducing the incidence of CPV. Future studies should investigate whether our findings can be extended to adolescents with ADHD who are not receiving medical treatment. Second, the temporal associations between CPV and other variables could not be determined because of the cross-sectional design of this study. Longitudinal studies are needed to examine the temporal correlations of CPV with the individual, parent-child interaction, and family factors. Third, although this study included ADHD symptom, externalizing problems such as ODD and conduct symptoms, and parental education level, the relationships of some factors such as medication type and socioeconomic status with CPV were not examined in this study. Further study including these factors is needed. Fourth, this study used only the information reported by adolescent and parents but no third-party or objective assessments. There might be informant bias. Fifth, given the number of predictors relative to events, there might be the risk of overfitting.

## Conclusion

Our results indicated that many adolescents with ADHD included in this study had engaged in CPV. Given that CPV can affect adolescent and parent health and damage parent-child relationships, engagement in CPV among adolescents with ADHD warrants regular evaluation and intervention. In this study, multiple individual factors (externalizing behavior problems and social information processing involving aggressive response), one parent-child interaction factor (parenting-to-child violence), and one family factor (violence among adult family members) were found to be significantly correlated with CPV. Overall, health-care professionals should incorporate these factors into their intervention programs to mitigate the risk of CPV among adolescents with ADHD. Additionally, parents should be included in such interventions to enhance their communication skills, reduce their aggressive discipline behavior, and mitigate the risk of CPV.

## Figures and Tables

**Table 1:** Demographic characteristics, behavioral problems, self-esteem, parenting styles, social information processing during parent-child conflict, and parent-to-child violence (N = 247)

	*n* (%)	Mean (SD)	Range
Adolescent gender			
Girl	41 (16.6)		
Boy	206 (83.4)		
Adolescent age (years)		13.2 (2.0)	11-18
Parent gender			
Female	182 (73.7)		
Male	65 (26.3)		
Parent age (years)		46.4 (6.4)	27-76
Parent educational level			
High school or lower	80 (32.4)		
College degree or higher	167 (67.6)		
Behavioral problems on the CBCL/6-18			
ADHD symptoms		61.9 (7.7)	50-80
Internalizing behavior problems		56.8 (10.2)	33-85
Externalizing behavior problems		55.7 (10.3)	33-78
Self-esteem on the RSES		19.2 (6.0)	3-30
Social information processing during parent-child conflict on the SIPCPC-Q			
Hostile attribution	121 (49.0)		
Anger	180 (72.9)		
Aggressive response	71 (28.7)		
Anticipation of positive consequences	14 (5.7)		
Empathy	108 (43.7)		
Parenting styles on the PBI			
Caring or affectionate parenting		38.9 (4.8)	23-48
Overprotective parenting		12.4 (3.0)	7-20
Authoritative parenting		11.7 (2.9)	6-20
Adolescent-reported parent-to-child violence	96 (38.9)		
Violence among adult family members	102 (41.3)		

ADHD: attention-deficit/hyperactivity disorder; CBCL/6-18: Child Behavior Checklist for Ages 6-18; PBI: Parental Bonding Instrument; RSES: Rosenberg Self-Esteem Scale; SIPCPC-Q: Social Information Processing in Child-to-Parent Conflicts Questionnaire.

**Table 2:** Types of and reasons for engagement in child-to-parent violence

	Adolescent- reported *n* (%)	Parent- reported *n* (%)	Adolescent- or parent-reported *n* (%)
Types of CPV (*N* = 247)			
Psychological aggression	94 (38.1)	95 (38.5)	137 (55.5)
Physical aggression	19 (7.7)	31 (12.6)	43 (17.4)
Financial demand	66 (26.7)	83 (33.6)	115 (46.6)
Control or domination	94 (38.1)	125 (50.6)	156 (63.2)
Any type	139 (56.3)	165 (66.8)	195 (78.9)
Reasons for adolescent engagement in CPV (*N* = 195)			
To be allowed to return home later after going out at night	19 (9.7)	16 (8.2)	32 (16.4)
To obtain more money from parents	42 (21.5)	43 (22.1)	72 (36.9)
To persuade parents to buy a desired item	56 (28.7)	74 (37.9)	104 (53.3)
To avoid chores	58 (29.7)	91 (46.7)	119 (61.0)
To avoid attending school or studying	57 (29.2)	69 (35.4)	98 (50.3)
In response to adolescent's anger	70 (35.9)	99 (51.8)	135 (69.2)
In response to parents' physical aggression	25 (12.8)	41 (21.0)	55 (28.2)
In response to parents' verbal aggression	47 (24.1)	71 (36.4)	92 (47.2)
In response to parental restrictions on 3C product use	67 (34.4)	106 (54.4)	136 (69.7)
In response to parents' differential treatment of their children	41 (21.0)	50 (25.6)	77 (39.5)
In response to parental restrictions on children's friendships or romantic relationships	12 (6.2)	11 (5.6)	22 (11.3)
In response to parental control of children's dressing style	31 (15.9)	20 (10.3)	46 (23.6)

CPV: Child-to-parent violence

**Table 3:** Factors correlated with child-to-parent violence: Bivariable logistic regression analysis

	Psychological aggression		Physical aggression		Financial demand		Control or domination	
	OR (95% CI)	*p*	OR (95% CI)	*p*	OR (95% CI)	*p*	OR (95% CI)	*p*
Adolescent gender^a^	0.763 (0.384, 1.512)	.438	4.845 (1.124, 20.897)	.034	1.011 (0.516, 1.979)	.976	1.811 (0.921, 3.562)	.085
Adolescent age	0.981 (0.867, 1.110)	.759	0.890 (0.748, 1.058)	.185	1.003 (0.887, 1.135)	.960	0.886 (0.780, 1.006)	.062
ADHD symptoms	1.082 (1.044, 1.123)	<.001	1.070 (1.026, 1.117)	.002	1.089 (1.050, 1.129)	<.001	1.099 (1.056, 1.144)	<.001
Internalizing behavior problems	1.057 (1.029, 1.086)	<.001	1.033 (1.000, 1.068)	.053	1.053 (1.025, 1.081)	<.001	1.075 (1.044, 1.107)	<.001
Externalizing behavior problems	1.076 (1.046, 1.106)	<.001	1.094 (1.052, 1.138)	<.001	1.088 (1.057, 1.120)	<.001	1.094 (1.061, 1.128)	<.001
Self-esteem	0.952 (0.912, 0.995)	.028	1.011 (0.956, 1.069)	.098	0.941 (0.901, 0.983)	.003	0.964 (0.922, 1.008)	.110
Hostile attribution on the SIPCPC-Q	1.574 (0.950, 2.610)	.078	1.755 (0.898, 3.431)	.100	1.447 (0.876, 2.392)	.149	1.703 (1.009, 2.874)	.046
Anger on the SIPCPC-Q	2.328 (1.313, 4.128)	.003	1.780 (0.780, 4.064)	.171	1.542 (0.871, 2.732)	.137	2.422 (1.364, 4.299)	.003
Aggressive response on the SIPCPC-Q	6.177 (3.104, 12.294)	<.001	2.609 (1.324, 5.138)	.003	1.367 (0.787, 2.376)	.267	5.201 (2.503, 10.808)	<.001
Anticipation of positive consequences on the SIPCPC-Q	11.427 (1.471, 88.782)	.020	1.316 (0.351, 4.932)	.684	2.157 (0.701, 6.631)	.180	1.490 (0.453, 4.895)	.511
Empathy on the SIPCPC-Q	0.994 (0.599, 1.648)	.980	0.696 (0.360, 1.345)	.696	0.782 (0.472, 1.295)	.340	1.089 (0.647, 1.833)	.748
Parent-to-child violence	6.757 (3.679, 12.410)	<.001	3.302 (1.668, 6.537)	.001	2.183 (1.296, 3.676)	.003	4.466 (2.418, 8.248)	<.001
Violence among adult family members	2.547 (1.498, 4.329)	.001	1.820 (0.938, 3.528)	.076	1.775 (1.064, 2.960)	.028	3.934 (2.186, 7.077)	.001
Caring or affectionate parenting	1.011 (0.959, 1.066)	.682	1.048 (0.975, 1.125)	.203	0.908 (0.859, 0.960)	.001	0.942 (0.890, 0.996)	.037
Overprotective parenting	1.038 (0.953, 1.130)	.391	0.970 (0.867, 1.085)	.596	1.004 (0.923, 1.093)	.923	1.143 (1.042, 1.253)	.003
Authoritative parenting	0.998 (0.914, 0.958)	.958	1.113 (0.988, 1.253)	.780	1.090 (0.997, 1.192)	.058	1.062 (0.970, 1.164)	.193

^a^Girls as a reference. ADHD: attention-deficit/hyperactivity disorder; SIPCPC-Q: Social Information Processing in Child-to-Parent Conflicts Questionnaire.

**Table 4:** Factors correlated with child-to-parent violence: Multivariable logistic regression analysis

	Psychological aggression		Physical aggression		Financial demand	Control or domination		
	OR (95% CI)	*p*	OR (95% CI)	*p*	OR (95% CI)	*p*	OR (95% CI)	*p*
ADHD symptoms	1.014 (0.957, 1.074)	.643	0.959 (0.895, 1.026)	.224	1.023 (0.971, 1.077)	.402	1.003 (0.491, 2.049)	.993
Internalizing behavior problems	1.015 (0.977, 1.054)	.444						
Externalizing behavior problems	1.032 (0.985, 1.082)	.180	1.107 (1.043, 1.176)	.001	1.065 (1.022, 1.110)	.003	4.073 (1.735, 9.564)	.001
Self-esteem					0.950 (0.904, 0.998)	.042		
Anger on the SIPCPC-Q	0.965 (0.830, 1.123)	.647					1.003 (0.491, 2.049)	.993
Aggressive response on the SIPCPC-Q	3.464 (1.526, 7.862)	.003	1.318 (0.954, 4.704)	.094			4.073 (1.735, 9.564)	.001
Parent-to-child violence	3.943 (2.008, 7.744)	<.001	2.237 (1.064, 4.704)	.034	1.338 (0.746, 2.402)	.329	2.260 (1.118, 4.566)	.023
Violence among adult family members	1.791 (0.950, 3.378)	.072					2.545 (1.303, 4.973)	.006
Caring or affectionate parenting					0.935 (0.879, 0.995)	.033		
Overprotective parenting							1.075 (0.962, 1.201)	.202

^a^Girls as a reference. ADHD: attention-deficit/hyperactivity disorder; SIPCPC-Q: Social Information Processing in Child-to-Parent Conflicts Questionnaire.
